# Anatomy of the splenic artery: what does the surgeon need to know?

**DOI:** 10.1590/0100-6991e-20223294-en

**Published:** 2022-09-21

**Authors:** DAVID MATHEUS VIANA DE MORAES, ATHANY GUTIERRES, RAMIRO COLLEONI, IVANA LORAINE LINDEMANN, ROBSON ROTTENFUSSER, JORGE ROBERTO MARCANTE CARLOTTO

**Affiliations:** 1 - Universidade Federal da Fronteira Sul, Departamento de Cirurgia - Passo Fundo - RS - Brasil; 2 - Universidade Federal de São Paulo, Disciplina de Gastroenterologia Cirúrgica - São Paulo - SP - Brasil; 3 - Hospital de Clínicas, Departamento de Cirurgia - Passo Fundo - RS - Brasil

**Keywords:** Splenic Artery, Anatomic Variation, Anatomy, Surgery, Computed Tomography, Artéria Esplênica, Variação Anatômica, Anatomia, Cirurgia, Tomografia Computadorizada

## Abstract

**Objective:**

to determine the prevalence and describe the main morphological and metric variations of the splenic artery in terms of its origin, path and polar and terminal branches.

**Methods:**

cross-sectional study, carried out at Hospital de Clínicas between July and November 2020. Computed tomography scans were analyzed with intravenous contrast of the patients seen at the Radiology and Diagnostic Imaging Service. The findings were categorized as to origin, path and splenic ramifications.

**Results:**

1,235 patients were evaluated. As for the origin, the splenic artery appears in the celiac trunk in 99.11% of the individuals. Of these, 5.95% have a bifurcated celiac pattern, 92.17% trifurcated and 1.88% tetrafurcated. The mean arterial diameter was 5.92mm (±1.2), the highest one being in white men. As for the path, the splenic artery was unique in the entire sample. The suprapancreatic course was found in 75.63% of the individuals, with a higher occurrence in women, 78.87% (p<0.001). The terminal splitting pattern of the splenic artery was characterized by the bifurcated type (95.47%). The terminal branches seen most frequently were those with three arteries (34.90%) and most individuals did not have polar branches.

**Conclusion:**

the splenic artery presents a highly variable pattern of origin and its average caliber is influenced by sex and color. The suprapancreatic path was the most characteristic and predominant in females. The bifurcated pattern of final division, with three terminal branches and the absence of polar arteries, occurs more frequently.

## RESUMO

**Objetivo:**

determinar a prevalência e descrever as principais variações morfológicas e métricas da artéria esplênica quanto a sua origem, trajeto e ramos terminais e polares.

**Métodos:**

estudo transversal, realizado no Hospital de Clínicas entre julho e novembro de 2020. Foram analisadas tomografias computadorizadas com contraste endovenoso dos pacientes atendidos no Serviço de Radiologia e Diagnóstico por Imagem. Os achados foram categorizados quanto à origem, trajeto e ramificações esplênicas.

**Resultados:**

foram avaliados 1.235 pacientes. Quanto à origem, a artéria esplênica surge no tronco celíaco em 99,11% dos indivíduos. Desses, 5,95% apresentam padrão celíaco bifurcado, 92,17% trifurcado e 1,88% tetrafurcado. O diâmetro arterial médio foi de 5,92mm (±1,2), sendo a maior média em homens brancos. Quanto ao trajeto, a artéria esplênica se apresentou única em toda a amostra. O curso suprapancreático esteve em 75,63% dos indivíduos, com maior ocorrência em mulheres 78,87% (p<0,001). O padrão de divisão terminal da artéria esplênica caracterizou-se pelo tipo bifurcado (95,47%). Os ramos terminais visualizados mais frequentemente foram os com três artérias (34,90%) e a maioria dos indivíduos não apresentou ramos polares.

**Conclusão:**

a artéria esplênica apresenta padrão de origem altamente variável e seu calibre médio é influenciado por sexo e cor. O trajeto suprapancreático foi o mais característico e predominou no sexo feminino. O padrão bifurcado de divisão final, com três ramos terminais e ausência de artérias polares, ocorre com maior frequência.

## INTRODUCTION

The splenic artery (SA) supplies the spleen and is responsible for part of the gastric and pancreatic blood input^1^. Its conventional emergence occurs in the abdominal portion of the aorta, at the level of the twelfth thoracic vertebra. At this point, the celiac trunk (CTR) gives rise to the branches of the left gastric artery (LGA), SA, and the common hepatic artery (CHA). The SA tortuously travels along the superior and posterior margin of the pancreas to the spleen. However, anatomical variations of the CTR may occur, including a bifurcated presentation, a trifurcated one different from the hepatogastrosplenic, a tetrafurcated, and even the absence of a trunk^2^. After its origin, the SA follows its course retroperitoneally, along the superior and posterior margins of the body and tail of the pancreas, following a horizontal and tortuous course to cross the splenorenal ligament towards the splenic hilum^3^. On its way, the artery gives off branches that carry blood supply to the stomach and pancreas and, finally, enters the splenorenal ligament, near the tail of the pancreas, where it divides into several branches, grouped into superior and inferior. Each terminal branch gives off small arteries that penetrate the splenic hilum. The branches that migrate to the upper or lower end of the spleen are called polar^3^ ^-^ ^5^.

The presentation of SA has variations in origin, course, ramifications, and anatomical relationships, and knowledge about the vascular pattern systematizes and facilitates the recognition of its anomalies^6^. The identification of such anomalies has gained importance since 1590, when Franciscus Rosetti performed the first partial splenectomy. The origin of SA, as well as its collateral and terminal branches, are factors to be considered, since they influence the surgical tactic to be used in gastric, pancreatic, splenic, and portal hypertension procedures^6^. The rare studies on anatomical variations and the imprecision of morphological and metric data of SA motivated this study, with the objective of determining the prevalence and describing the main morphological and metric variations of the SA origin, course, and its terminal and polar branches.

## METHODS

In compliance with Resolution 466/2012 of the National Health Council, the study protocol was approved by the Ethics Committee in Research with Human Beings of the Universidade Federal da Fronteira Sul. The study was exempted from the Free and Informed Consent Form (Registration number 3,929,428).

The study is characterized as cross-sectional and was carried out at Hospital de Clínicas de Passo Fundo (HCPF), State of Rio Grande do Sul, Brazil. The non-probabilistic sample was selected for convenience and consisted of patients of any age and both sexes, seen between August 1 and December 31, 2018, at the Radiology and Diagnostic Imaging Service of the HCPF. Considering an expected prevalence of 10% of alterations in the origin of the splenic artery and a confidence interval of 95%, the estimated sample size would be 139 participants. However, to increase results’ accuracy, we decided to include a greater number of patients, defining the population according to a determined period of patient care.

Thus, for the composition of the sample, the radiology service provided a list with 2,000 codes referring to patients. From this list, we excluded duplicated codes, as some patients underwent more than one CT scan in the period. Secondly we excluded CT scans of patients with a previous history of splenic surgery, in addition to those that did not use intravenous contrast and those that contained alterations that made it impossible to perform morphometric measurements.

Initially, we sought electronic medical records (PEP Software, MV2000) to exclude patients with a history of previous splenic surgery and to access demographic information. After this screening, we performed the tomographic evaluation using the Picture Archiving and Communication System (PACS^®^) Arya Aurora (Pixeon^®^, 2020). Through CT scans, we obtained the diameter of the splenic artery at its origin, the place of origin, course, number of splenic arterial branches, and polar arteries. Two physicians specialized in abdominal radiology carried out the evaluation, which were double-typed and validated using the EpiData^®^ open-source software, version 3.1.

Statistical analysis included descriptive measurements and verification of the frequency distribution of the type of splenic artery course according to sex (Pearson chi-square, assuming an α error of 5%). Moreover, we compared the mean diameters at the SA origin according to sex and skin color (Student’s t test, α error of 5%). All analyzes were performed with the PSPP^®^ open-source software.

## RESULTS

The sample consisted of 1,235 participants, who were predominantly male (50.53%), white (93.96%), and aged between 60 and 69 years (24.21%).

The origin of the SA was observed from some conformation of the CTR in 99.11% of the sample. In the remaining, we observed direct emergence from the abdominal aorta (AA) in 0.65%, from the CHA in 0.16%, and from the superior mesenteric artery (SMA) in 0.08%. Of those originating from the CTR, 5.95% had a bifurcated celiac pattern, 92.17% trifurcated, and 1.88% tetrafurcated (Table 1).

**Table 1:** Características da artéria esplênica.

Variables	n	%
Origin		
Celiac trunk	1,224	99.11
Abdominal aorta	8	0.65
Superior mesenteric	1	0.08
Common hepatic	2	0.16
Type of celiac origin		
Bifurcated	73	5.95
Variables	n	%
Trifurcated	1,130	92.17
Tetrafurcated	23	1.88
Course		
Single	1,235	100
Course type		
Suprapancreatic	934	75.63
Dorsal	301	24.37
Final branching pattern		
Bifurcated	1,179	95.47
Trifurcated	56	4.53
Terminal splenic branches		
1	2	0.16
2	135	10.93
3	438	34.90
4	428	34.10
5	176	14.02
6	42	3.40
7	11	0.89
8	3	0.24
Polar splenic branches		
0	1,113	90.12
1	113	9.15
2	8	0.65
3	1	0.08

**Figure 1:**
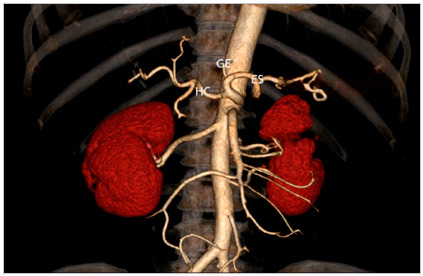
Three-dimensional reconstruction demonstrating the celiac trunk with usual branching into the left gastric (LGA), splenic (SA) and common hepatic (CHA) arteries.

In those with trifurcated trunks, the classic presentation, formed by the common, splenic, and left gastric hepatic arteries (Figure 1) occurred in 98.94.

**Figure 2:**
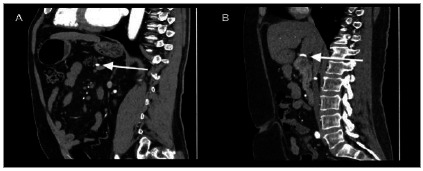
Computed tomography sagittal plane reconstructions demonstrating (A) dorsal course (white arrow) and (B) suprapancreatic course (white arrow) of the splenic artery.

The SA course was single in the entire sample, and regarding the course, it was classified as suprapancreatic and dorsal (Figure 2), being suprapancreatic in 75.63% and dorsal in 24.37% individuals (Table 1). 

Table 2 shows the frequency of the SA course type according to patients’ sex. There was a predominance of the suprapancreatic course in relation to the dorsal course in both sexes, with the suprapancreatic distribution being more frequent in females compared with males (78.87% vs. 71.47%) and the dorsal course was more predominant in men (28.53% vs. 20.13%), p=0.001.

**Table 2:** Type and distribution of splenic artery course by sex.

	Suprapancreatic		Dorsal		p
Variable	n	%	n	%	
Sex					0.001
Male	446	71.47	178	28.53	
Female	488	78.87	123	20.13	

*Chi-square test.

**Figure 3:**
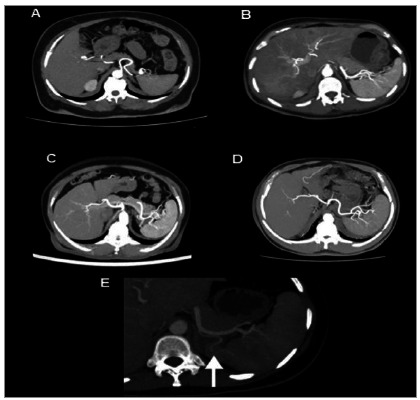
Maximum Intensity Projection (MIP) reconstructions in the axial computed tomography plane demonstrating (A) two terminal branches, (B) three terminal branches, (C) four terminal branches, (D) five terminal branches, and (E) polar artery (white arrow).

Regarding the splenic branches, the terminal arteries presented as one artery in 0.16% of the individuals, two in 10.93%, three in 34.90%, four in 34.10%, five in 14.02%, six in 3.40%, seven in 0.89%, and eight in 0.24%. We found polar splenic branches in 9.88% of the sample. We observed a single polar branch in 9.15% of the individuals, two in 0.65%, and three in 0.08%. That is, the most common terminal patterns were those that presented between two and five arteries and, among those that presented polar branches, a single branch was the most common (Table 1). Figure 3 shows the images of the most frequently observed patterns.

As for the metric findings, the mean diameter of the splenic artery at its origin was 5.92mm (±1.2), with a range of 2.81mm to 10.84mm. There was also a statistically significant difference in diameter variation in relation to sex and race. We found a higher mean diameter in males (6.16mm ±1.17; p<0.001) and in whites (5.93mm ±1.14; p=0.041) (Table 3).

**Table 3:** Diameter of the splenic artery at its origin by sex and race.

Diameter (mm)			
Variable	n (%)	Mean (±SD)	p
Sex			<0.001
Male	624 (50.53)	6.16 ± 1.17	
Female	611 (49.47)	5.67 ± 1.07	
Race (n=1,226)			0.041
White	1.152 (93.96)	5.93 ± 1.14	
Others	74 (6.04)	5.66 ± 1.05	

SD: standard deviation; *Student ‘s t test.

## DISCUSSION

### Origin

The exact identification of the splenic artery’s origin, as well as its metric characteristics, is essential not only for surgical planning, but also to avoid iatrogenic injuries during surgery. Thus, knowledge of its origin and diameter is essential in gastrointestinal operations and, especially, in the application of stents in endovascular surgery^7^. During embryogenesis, each metameric level gives rise to three pairs of arteries from the aorta. The posterior ones are parietal, the lateral ones are urogenital, and the anterior ones are intestinal. According to Tandler et al., the intestinal arteries are connected by an anterior longitudinal anastomosis (laengsanastomosis)^8^. Of the four arterial roots, two disappear and the remaining anastomoses give rise to the CHA, SA, and LHA^9^. The exacerbated growth or regression of these vessels is related to the occurrence of variations in the celiac trunk and in the superior mesenteric artery.

Méndez found that the emergence of SA from the CTR occurs in 88.46%^5^. Clement et al., in a survey with cadavers and imaging exams, observed 90.5% of occurrence of the classic pattern of trifurcation^10^. According to Song et al., the CTR classic presentation occurs in 89.1%^1^ ^1^. In the present study, the splenic artery presented celiac origin in 99.11% of the patients and the classic presentation of the hepatogastrosplenic trunk occurred in 91.47%. The observation of higher frequencies may be due to the differences between the studied samples, especially regarding sample size and ethnic origin of the participants, in addition to the particularities of the methods used in the studies.

The SA emergence from the abdominal aorta occurred only in 0.65% of the sample, from the common hepatic artery in 0.16%, and from the superior mesenteric artery in 0.08%. These findings are similar to those of Indian studies^12^ ^-^ ^13^. The bifurcated celiac trunk, in different combinations, has been observed in up to 12% of the population^1^ ^2^. In this study, CTR bifurcation was found in 5.95% of the sample.

The metric assessment of the splenic artery diameter at its origin resulted in an average of 5.92mm (± 1.2), with a variation from 2.81mm to 10.84mm. Méndez found that the mean SA length was 147mm, with a range from 81mm to 306mm and that its mean diameter was 8.6mm, with a range of 4 to 11mm^5^. In this study, male subjects had a higher mean arterial diameter than females (6.16 ± 1.17 : 5.67 ± 1.07), and white subjects had a higher mean than other races (5.93 ± 1.14 : 5.66 ± 1.05). We found no studies in the literature that evaluated the relationship between these variables.

### Course

The course of SA can be classified as having two main patterns: the suprapancreatic course, where the artery curves and runs superior to the pancreas, and the dorsal course, where it runs relatively straight to the dorsal side of the pancreas. We observed a splenic artery single course throughout the sample, with no occurrence of its duplicated form. We found a suprapancreatic course in 75.63% and a dorsal one in 24.37% of the individuals. Such findings are in line with a study carried out on 320 cadavers, that recorded a suprapancreatic path in 74.1%^1^. Although we found no previous reports in the literature on the course type according to sex, we observed that the suprapancreatic path was more frequent in both sexes in our sample. However, when comparing the sexes within each pattern, there was a predominance of females and males, respectively, in the suprapancreatic and dorsal courses.

Knowledge of the path can influence the surgical technique used. The study carried out by Inoko et al. demonstrated that the safety of laparoscopic splenic-preserving distal pancreatectomy is associated with a previous study of the SA course and terminal branches^15^. The surgical technique used was decided according to the arterial path, and in cases where the course was suprapancreatic, the peritoneal incision was performed along the pancreatic border. In cases where the SA course was dorsal, the artery was exposed and protected by the inferior approach^15^ ^-^ ^19^.

### Branching

Knowledge of the intraparenchymal division of the splenic artery is essential in partial splenectomy. Therefore, understanding the vascular network presentation facilitates its rapid identification and characterizes an important parameter for surgical guidance^20^.

The pattern of terminal division of the splenic artery was mostly characterized by the bifurcated type (95.47%). The trifurcated presentation was present in 4.53%. The findings are similar to those observed in a Brazilian study with 60 arteriographies, in which the bifurcated type appeared in 93.3%, and the trifurcated, in 6.7%^20^. Regarding terminal splenic ramifications, the most frequently found were those of three (34.90%) and four arteries (34.10%). A single branch occurred in 0.16% of the cases. In the observations carried out in an Indian study, the splenic artery divided into terminal branches in 97.2% and, in the remaining cases, it entered the splenic hilum without branching. Also in the same study, the authors identified a higher frequency of two (63.1%) and four (18.8%) terminal branches^1^. The absence of splenic polar arteries predominated in the sample. A single polar artery was present in 9.15%, two in 0.65%, and three in only 0.08% of the individuals.

The main strengths of this study include the detailed description of the anatomical course of the splenic artery, the variation in arterial diameter according to sex and race, as well as the difference observed in the type of course according to sex. In this context, it is important to emphasize that the analysis of arterial diameter, when performed, is carried out in cadavers, with a reduced sample number, or even in patients with arteries affected by some disease. In this case, our study included a sample of more than 1,200 cases, with arteries without clinical involvement. It is also noteworthy that the observation of a higher frequency of the suprapancreatic course in both sexes and a greater frequency of the dorsal course in men is unprecedented in the literature.

## CONCLUSION

The splenic artery has a highly variable pattern of origin, and its average caliber is influenced by patients’ sex and skin color. Its course is mostly suprapancreatic and is even more common in females. The bifurcated pattern of final division, with three terminal branches and absence of polar arteries, was the most frequent.
